# Epstein-Barr Virus-Positive Primary Diffuse Large B-cell Lymphoma of the Colon in a Patient With Human Immunodeficiency Virus

**DOI:** 10.7759/cureus.31046

**Published:** 2022-11-03

**Authors:** Nyan A Bethel, Sindhusha Veeraballi, Sahithi Chittamuri, Hamid Shaaban, Gunwant Guron

**Affiliations:** 1 Internal Medicine, Saint Michael's Medical Center, Newark, USA; 2 Hematology and Oncology, Saint Michael's Medical Center, Newark, USA

**Keywords:** non-hodgkin's lymphoma, epstein-barr virus, hiv, ascending colon, diffuse large b-cell lymphoma

## Abstract

Colorectal lymphomas of primary origin are rare neoplasms accounting for 3% of all lymphomas involving the gastrointestinal (GI) tract, and of that 3%, 0.1-0.5% involve the colorectal region. Among the types of non-Hodgkin's lymphomas involving the GI tract, diffuse large B-cell lymphoma (DLBCL) is the most common type. While extranodal involvement of DLBCL in the GI is common, DLBCL of the colon with Epstein-Barr virus positivity is a rare entity with only a few cases reported in the literature. Here, we present a rare case of a 53-year-old female with human immunodeficiency virus (HIV) who presented with generalized abdominal pain, weight loss, night sweats, and fevers. A computed tomography scan of the abdomen and pelvis showed a mass on the right side of the colon with associated retroperitoneal and right inguinal lymphadenopathy. She later underwent a colonoscopy with a biopsy. Histopathology showed DLBCL of the ascending colon and chemotherapy was initiated.

## Introduction

Diffuse large B-cell lymphoma (DLBCL) is the most common type of non-Hodgkin’s lymphoma (NHL) amounting to approximately 24% of all NHL cases in America annually [1]. Colorectal lymphomas of primary origin are uncommon in the GI tract accounting for approximately 3%, with 0.1-0.5% involving the colorectal region [2]. DLBCL by itself is aggressive. It becomes even more aggressive with concurrent Epstein-Barr virus (EBV) positivity and is characterized by rapidly progressing lymphadenopathy, fever, weight loss, and night sweats (B symptoms) [3]. Diagnosis is made by excisional lymph node biopsy with immunophenotypic analysis as well as bone marrow aspirate and biopsy needed more so for staging [3,4]. The basis of treatment entails surgical resection preceded by chemotherapy consisting of cyclophosphamide, hydroxydaunorubicin, oncovin, and prednisone (CHOP) with or without rituximab [5,6]. Patients receiving this regimen are at increased risk of perforation, with the median day being 46 [7]. Herein, we present a case of primary EBV-positive DLBCL of the ascending colon in a patient with well-controlled HIV who presented with abdominal pain and B symptoms.

## Case presentation

A 53-year-old African American female with a past medical history of well-controlled HIV with Symtuza and diabetes mellitus presented to the emergency department with complaints of diffuse abdominal pain, associated with bloody stools, fevers, night sweats, and weight loss. Her vitals were stable. On physical examination, there were right cervical and right inguinal lymphadenopathy. There was tenderness to palpation in all four quadrants without signs of guarding or rigidity and bowel sounds were normal.

**Figure 1:**
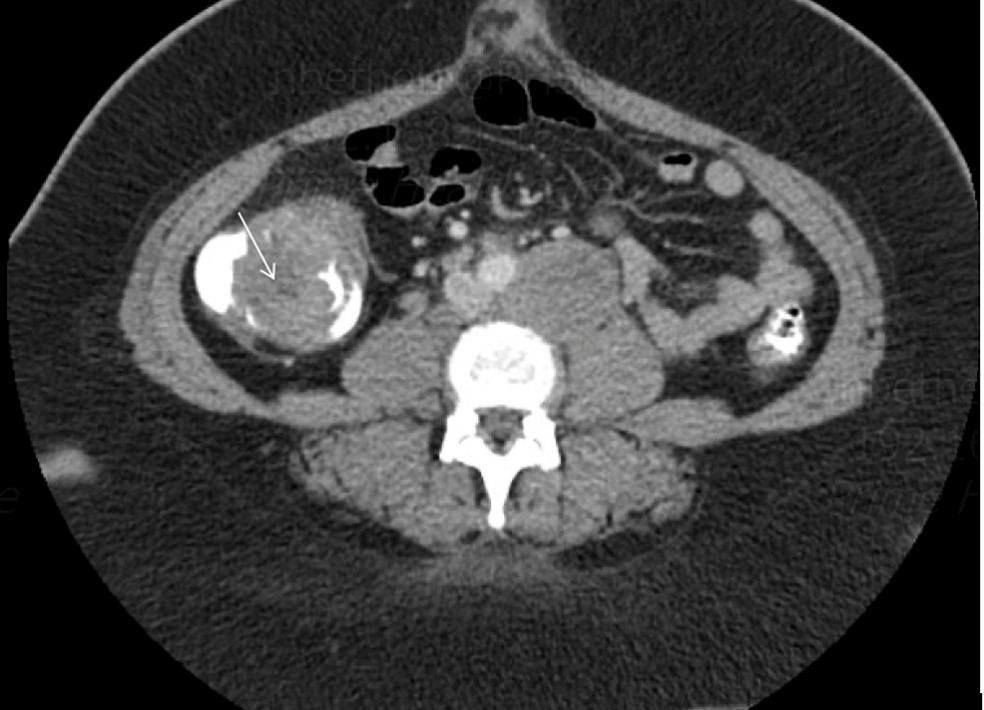
Axial computed tomography showing ascending colonic mass (white arrow)

**Figure 2:**
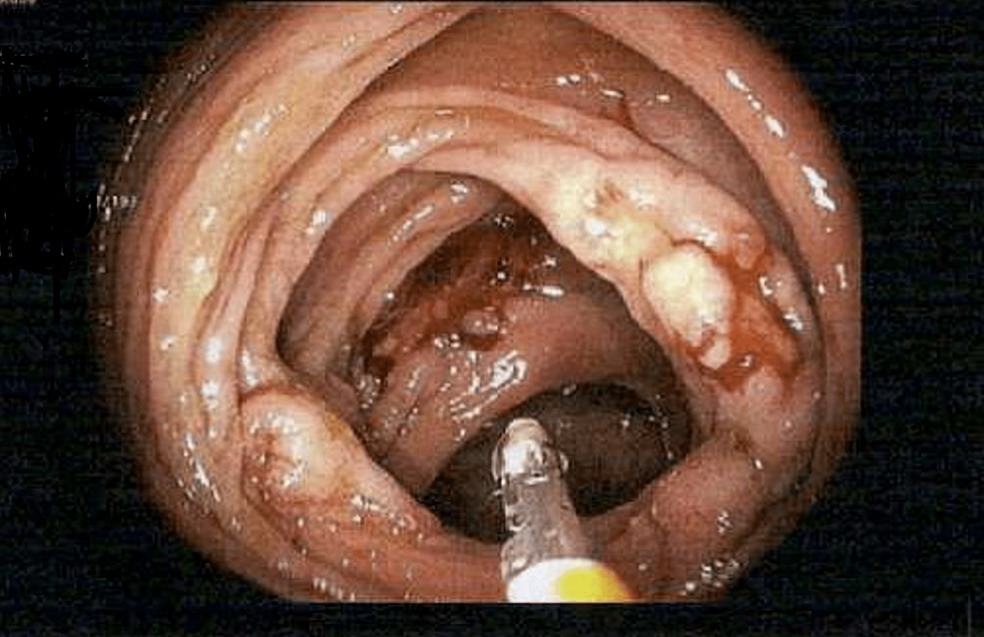
Colonoscopy demonstrating mass

**Figure 3:**
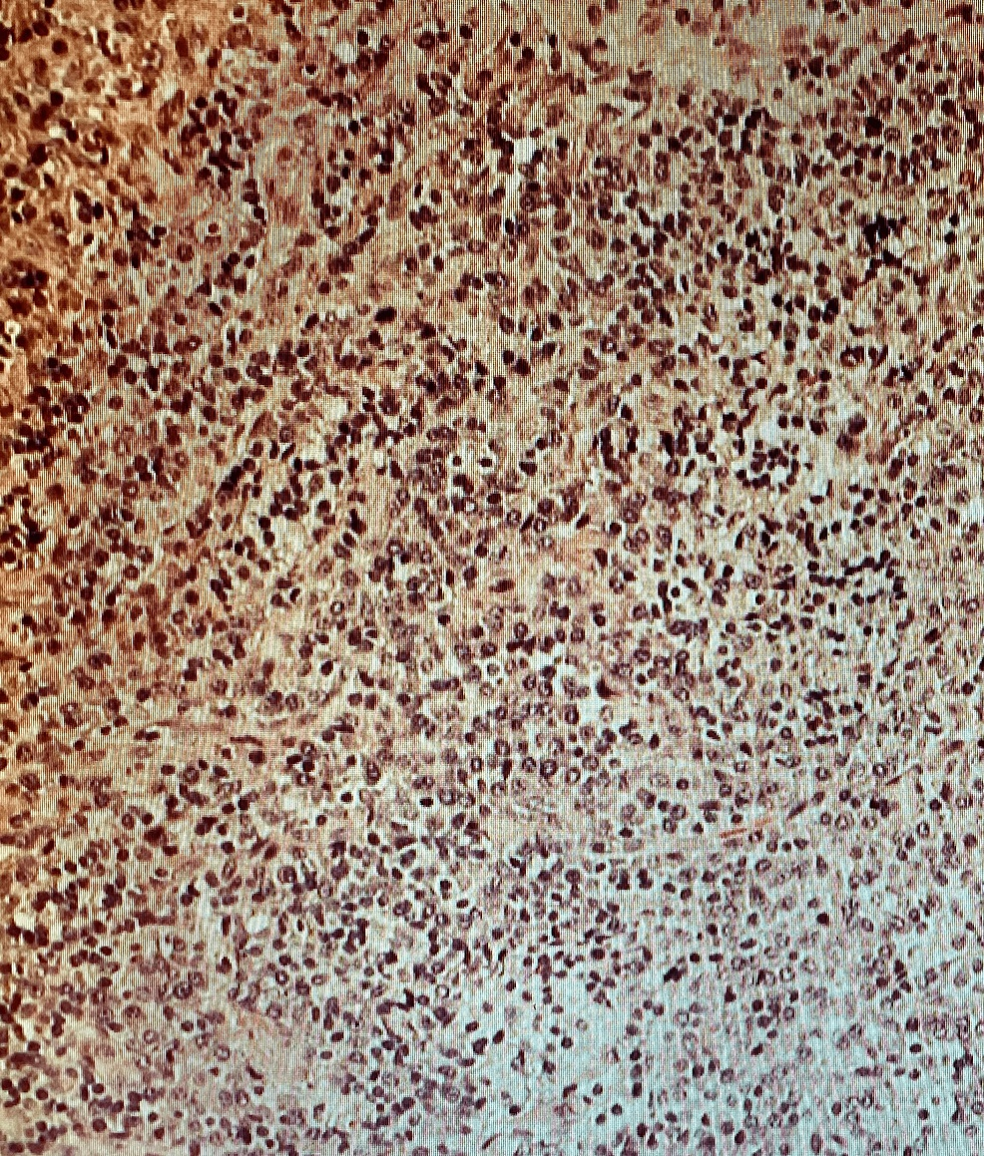
Hematoxylin & eosin stain demonstrating abnormal population of B cells (x100)

**Figure 4:**
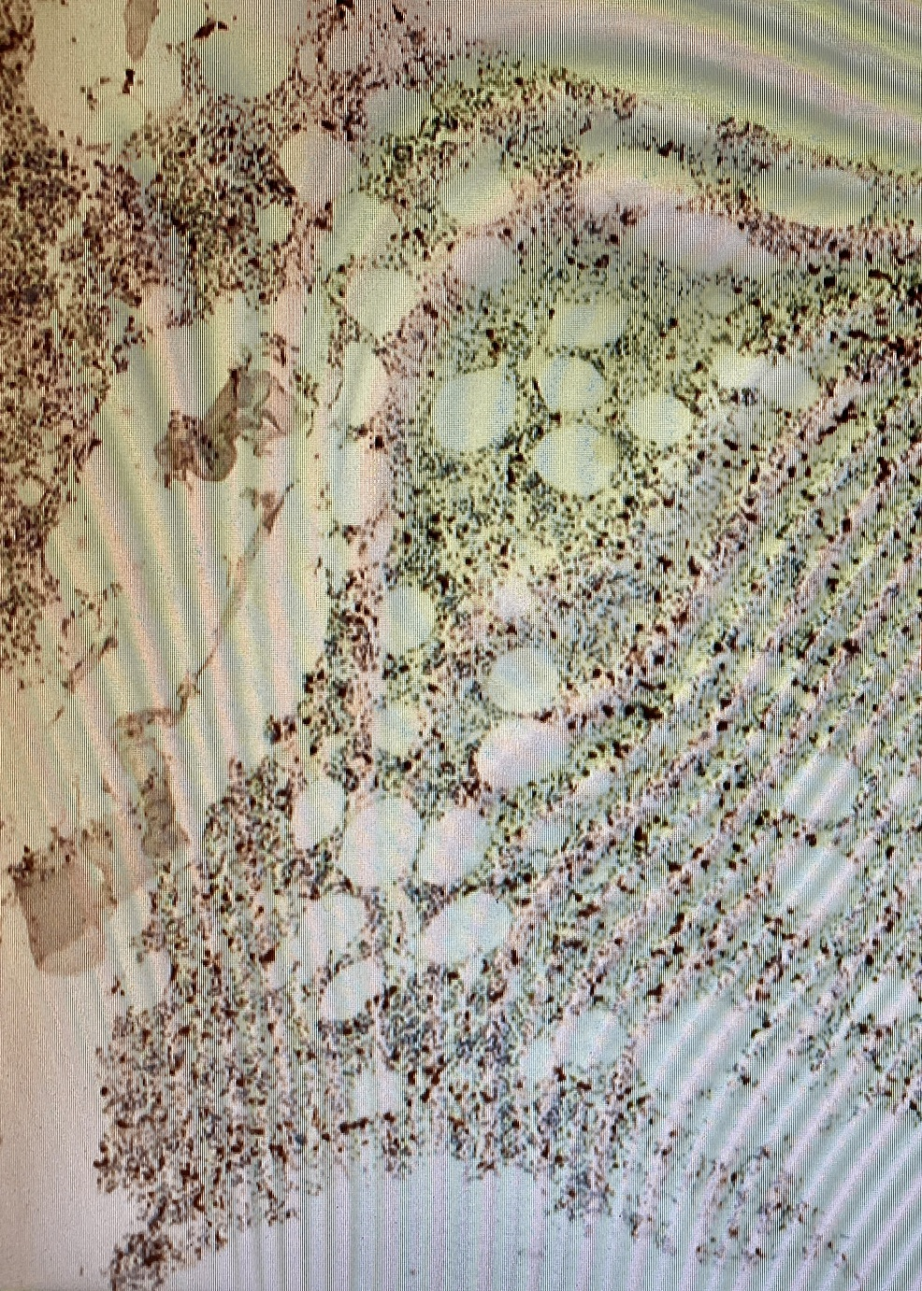
CD79a immunohistochemical stain showing B cells (x40)

**Figure 5:**
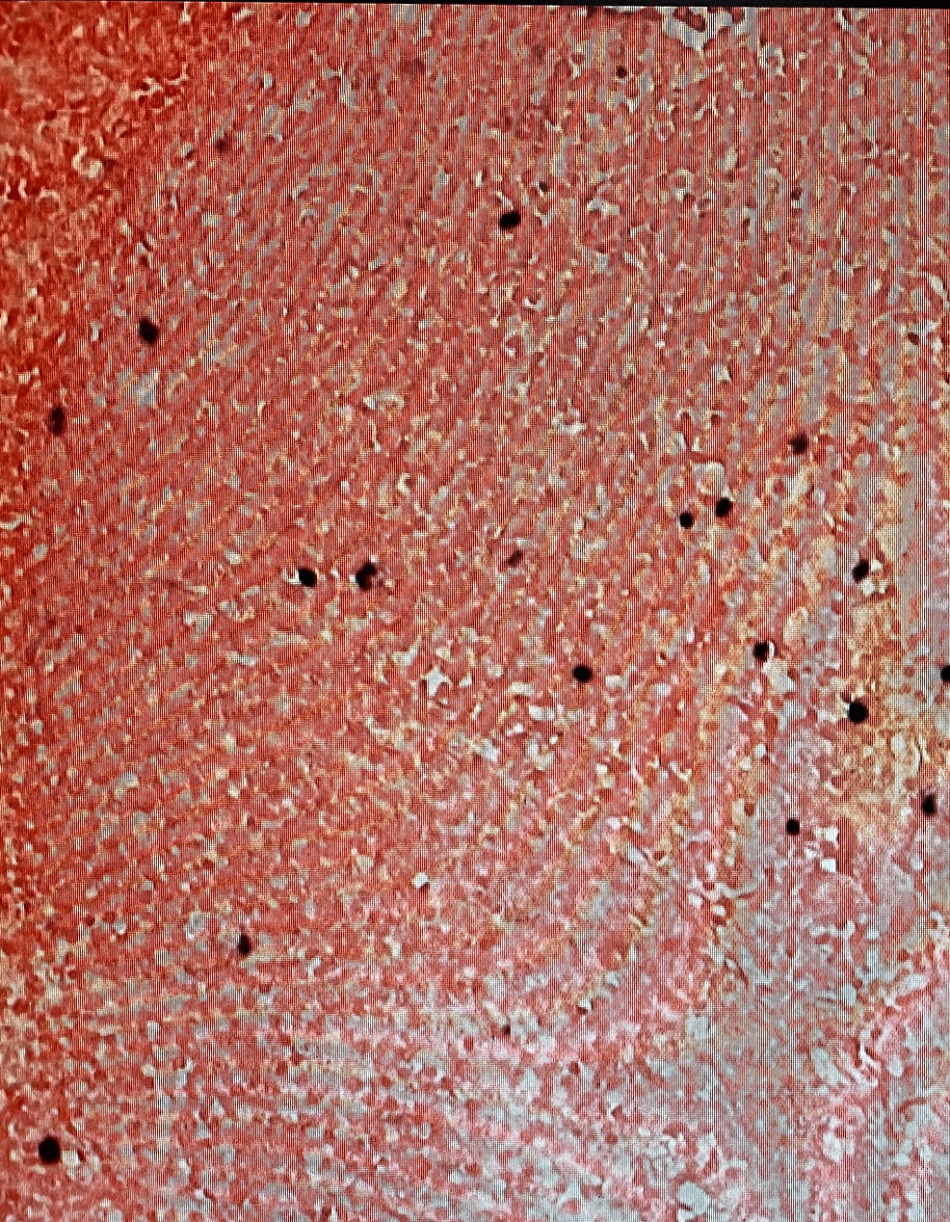
Epstein-Barr virus-positive infected B cells (x100)

**Figure 6:**
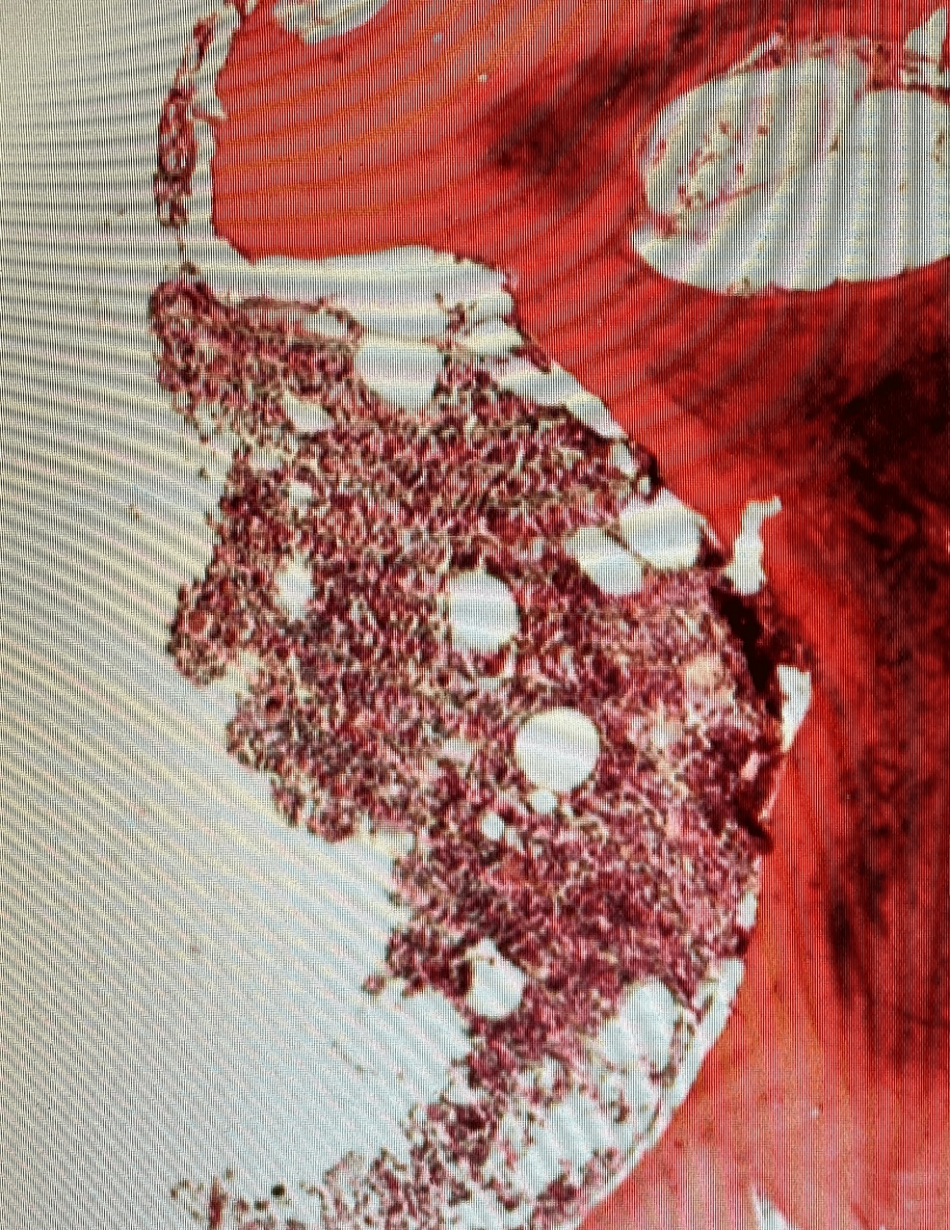
Bone marrow (x40)

**Figure 7:**
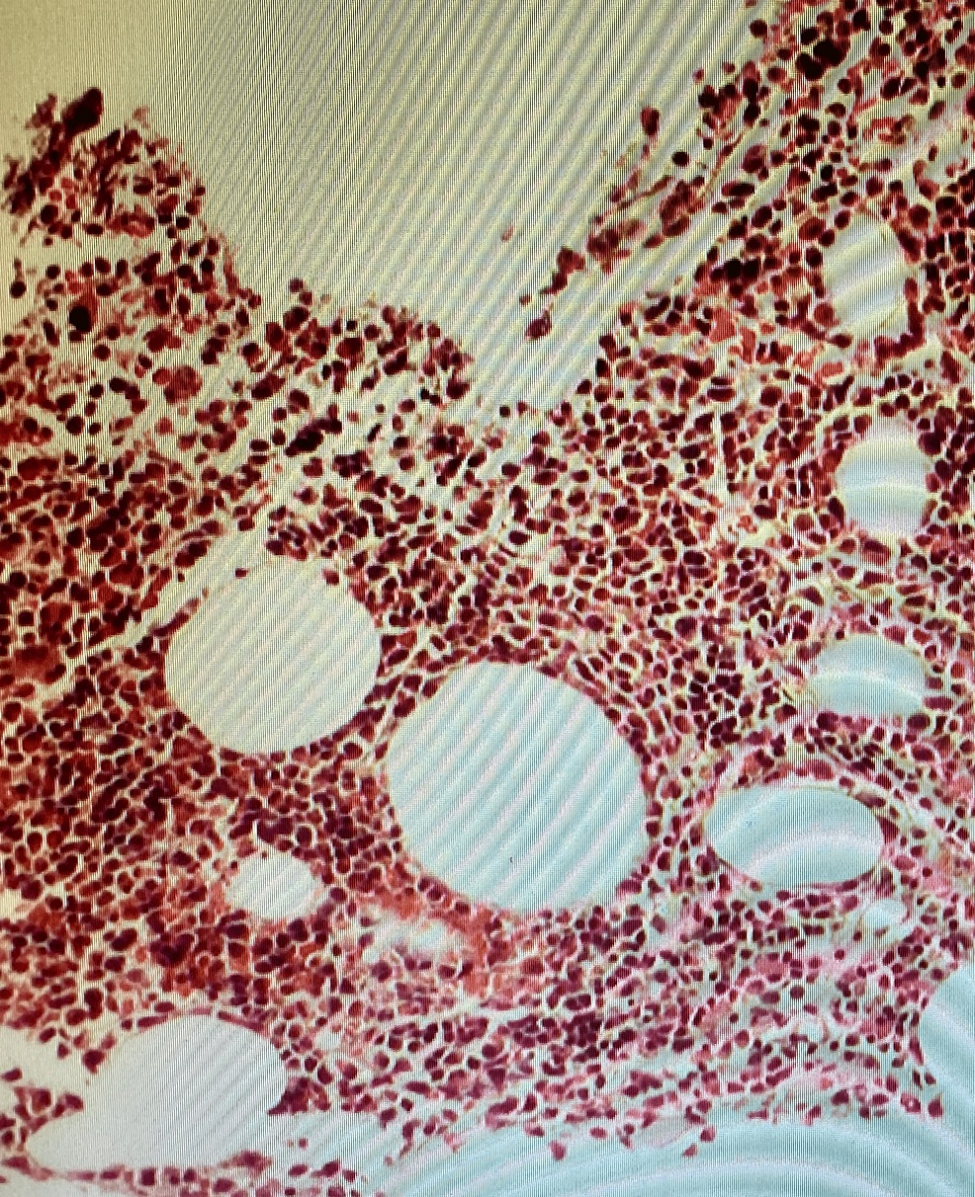
Clusters of lymphocytes admixed with lymphocytic cells (x100)

**Figure 8:**
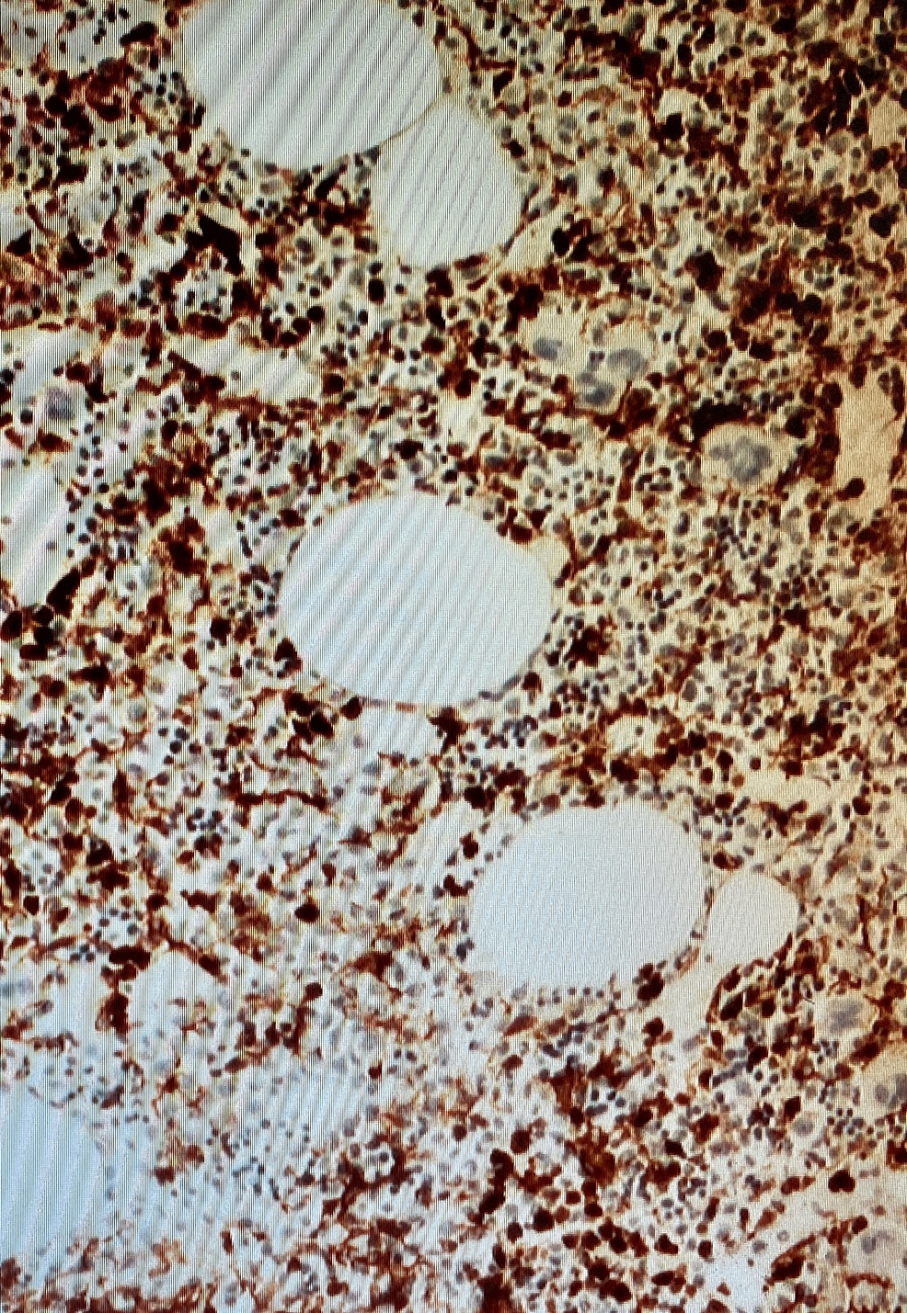
Numerous cluster (CD10+) lymphocytes (x100)

Laboratory studies were normal except for lactate dehydrogenase (LDH) of 1883. Human T-lymphotropic virus (HTLV) and hepatitis C antibodies were negative. CT scan of the abdomen and pelvis showed diffuse retroperitoneal and pelvic lymphadenopathy and a large eccentric mass measuring 5.4 x 2.8 cm in the right hemicolon (Figure 1). Surgery, oncology, and gastroenterology were consulted. The patient underwent exploratory laparotomy with a biopsy of the right inguinal lymph node and colonoscopy with resection of the mass. Colonoscopy showed multiple areas of frog-like/villous and sessile non-obstructing polypoid mass in the ascending colon (Figure 2) and multiple areas of erythema in the cecum. Pathology of the lymph node showed atypical clonal B-cell proliferation (Figure 3), which stained positive for CD79a (Figure 4). EBV-encoded small RNA (EBER) stain was positive (Figure 5). Biopsy of the cecum and ascending colon showed atypical lymphocytic infiltrate. Immunostains showed atypical lymphocytes with a Ki-67 proliferation rate of 60% and were positive for CD20, PAX-5, CD10, B-cell lymphoma 6 (BCL-6), and C-MYC, and negative for B-cell lymphoma 2 (BCL-2) with a high proliferation rate of Ki-67. Fluorescence in situ hybridization (FISH) was positive for c-MYC. Bone marrow biopsy was obtained (Figure 6) and showed numerous clusters of CD10+ lymphocytes (Figures 7, 8). Based on these findings, a diagnosis of stage IV DLBCL was confirmed.

Based on the International Prognostic Index for Diffuse Large B-cell Lymphoma score of 3, she was at high intermittent risk. She received 1 cycle consisting of rituximab, cyclophosphamide, hydroxydaunorubicin, Oncovin, and prednisone (R-CHOP). Approximately one week later, she presented to the ED with fevers, chills, generalized body pain, and abdominal pain. She was tachycardic and hypotensive. A complete blood count (CBC) showed an absolute neutrophil count of 0. Blood and urine cultures were obtained. She was admitted to the intensive care unit for febrile neutropenia with shock and was started on intravenous hydration, meropenem, Zosyn, and vancomycin. Chest X-ray and CT scan of the chest, abdomen, and pelvis were obtained. Bowel perforation was ruled out. She expired 18 hours later. Blood cultures eventually grew pan-sensitive pseudomonas bacteremia.

## Discussion

NHL is a form of malignant neoplasm that originates from T lymphocytes, B lymphocytes, and natural killer cells [8]. Of the many subtypes of B cell lymphomas, DLBCL is the most common. Other subtypes include Burkitt’s lymphoma, mantle cell lymphoma, and follicular lymphoma. When the colon is involved, studies show that there is a male predominance with the onset in the 6th to 7th decade of life [8-10]. More so, predisposing factors have not been identified; however, some literature states that celiac disease [8], immunosuppression, HIV, and inflammatory bowel disease have increased incidence in the development of this neoplasm [2,10].

In patients diagnosed with DLBCL, efforts should be made to obtain the EBV status of the patient, since it is the determining factor in treatment response [11-13]. EBV-positive (EBV+) DLBCL, not otherwise specified (NOS) according to the WHO classification, is a form of lymphoid neoplasm that is aggressive and is associated with chronic EBV infection [11]. EBV+ DLBCL is present in approximately 8-15% of DLBCL in the Asian population and <5% in the Western population [10,12], as evident in our patient. It has a poor prognosis and poor response to standard chemotherapeutic regimens [11-13]. Oyama et al. evaluated the chemotherapeutic response in 96 and 107 patients with EBV+ and EBV-negative DLBCL, respectively [14]. These patients were administered an anthracycline-based regimen and observed over time. It showed that the EBV-negative group had a response rate of 99%, whereas the EBV+ group had a response rate of 80%, thus suggesting a positive correlation between EBV-negative DLBCL and treatment response. Furthermore, according to Murthy et al., EBV+ DLBCL usually has a poor response to treatment [10], with a two-year median survival [15].

Patients with DLBCL typically express pan B cell markers like CD19, CD20, CD22, CD45, and CD79a, and BCL-6 protein is expressed in 70% of cases while CD10 is expressed in 30-60% of cases [2,5]. Ki-67 is also seen in about 70% of cases [12]. In our case, the cells were positive for CD20, CD10, BCL-6, PAX-5, and C-MYC. BCL-2 was negative. EBER was also positive, indicating the presence of EBV. EBV is more so seen in a different subcategory of B-cell lymphoma known as Burkitt’s lymphoma [13]; however, it was identified in our patient with DLBCL and is thus a very rare association.

CT is the initial diagnostic method that aids in the identification of any localized mass as well as associated lymphadenopathy. For diagnosing primary lymphoma of the colon, colonoscopy is the test of choice for evaluating the morphology as well as obtaining a biopsy [16]. The gold standard in diagnosis is biopsy [17]. Primary lymphomas of the colon typically involve the submucosa, thus making obtaining an adequate specimen difficult and necessitating the need for experienced endoscopists [17].

The International Prognostic Index (IPI) score is the most common tool utilized in estimating the outcome in patients with DLBCL who are receiving treatment. It takes into account the patient’s age, Ann Arbor stage, performance status, serum LDH, and extranodal site involvement. The calculated IPI score in our patient was 3, which puts her at high intermittent risk. In recent times, a new evolving technique known as gene profiling has been used [2], but the IPI score remains the most frequently used.

Management of DLBCL usually includes chemotherapy, surgery, radiotherapy, or combination therapy. However, the role of surgery is debatable [16]. Cai et al. reported the site-dependent efficacy of surgery and improved survival with surgical intervention in right-sided primary colonic lymphomas when compared to the left side and rectum. The gold standard treatment for DLBCL has always been chemotherapy. R-CHOP regimen has shown efficacy in survival benefits. Several other combination therapies and targeted therapies are under investigation. EBV+ DLBCL usually has a poor response to treatment [10], with a two-year median survival [15]. Treatment with the R-CHOP regimen especially when the GI tract is involved poses the risk of perforation. Vaidya et al. mentioned that 59% of the small bowel, 22% of the colon, and 16% of the gastric region were the common sites involved in perforation with the median day being 46 [7].

## Conclusions

Lymphomas involving the colorectal region are rare, with the most common type being DLBCL. Immunohistopathology remains the key to the diagnosis. The mainstay of treatment involves surgery with combination chemotherapy consisting of CHOP with or without rituximab. Since treatment outcome is strongly based on EBV positivity, it is imperative to evaluate the EBV status in every patient diagnosed with DLBCL. More research into treatment options for this unique subtype of DLBCL is needed to improve overall survival.

## References

[1] Liu Y, Barta SK (2019). Diffuse large B-cell lymphoma: 2019 update on diagnosis, risk stratification, and treatment. Am J Hematol.

[2] Barbaryan A, Ali AM, Kwatra SG (2013). Primary diffuse large B-cell lymphoma of the ascending colon. Rare Tumors.

[3] Sapkota S, Shaikh H (2022). Non-Hodgkin Lymphoma. https://www.ncbi.nlm.nih.gov/books/NBK559328/.

[4] Haddad I, El Kurdi B, El Iskandarani M, Babar S, Young M (2019). Primary diffuse large B-cell lymphoma of the sigmoid colon. Cureus.

[5] Modi V, Bajaj N, Lakkasani S, Shaaban HS, Guron G (2020). A rare and unusual presentation of Epstein-Barr virus-associated diffuse large B-cell lymphoma involving colon as the primary site. J Cancer Res Ther.

[6] Chen L, Sun Q, Chen E, Jin D, Song Z (2021). Primary colonic lymphoma: report of two cases and a literature review. J Int Med Res.

[7] Vaidya R, Habermann TM, Donohue JH (2013). Bowel perforation in intestinal lymphoma: incidence and clinical features. Ann Oncol.

[8] Erginoz E, Askar A, Cavus GH, Velidedeoglu M (2021). Primary diffuse large B-cell lymphoma of the sigmoid colon. Int J Surg Case Rep.

[9] Tahir M, Samad K, Koenig T, Viswanathan P (2018). A rare case of primary diffuse large B-cell lymphoma of the colon. AME Case Rep.

[10] Murthy SL, Hitchcock MA, Endicott-Yazdani TR, Watson JT, Krause JR (2017). Epstein-Barr virus-positive diffuse large B-cell lymphoma. Proc (Bayl Univ Med Cent).

[11] Beltran BE, Castro D, Paredes S, Miranda RN, Castillo JJ (2020). EBV-positive diffuse large B-cell lymphoma, not otherwise specified: 2020 update on diagnosis, risk-stratification and management. Am J Hematol.

[12] Gibson SE, Hsi ED (2009). Epstein-Barr virus-positive B-cell lymphoma of the elderly at a United States tertiary medical center: an uncommon aggressive lymphoma with a nongerminal center B-cell phenotype. Hum Pathol.

[13] Castillo JJ, Beltran BE, Miranda RN, Paydas S, Winer ES, Butera JN (2011). Epstein-Barr virus-positive diffuse large B-cell lymphoma of the elderly: what we know so far. Oncologist.

[14] Oyama T, Yamamoto K, Asano N (2007). Age-related EBV-associated B-cell lymphoproliferative disorders constitute a distinct clinicopathologic group: a study of 96 patients. Clin Cancer Res.

[15] Ok CY, Papathomas TG, Medeiros LJ, Young KH (2013). EBV-positive diffuse large B-cell lymphoma of the elderly. Blood.

[16] Stanojevic GZ, Nestorovic MD, Brankovic BR, Stojanovic MP, Jovanovic MM, Radojkovic MD (2011). Primary colorectal lymphoma: an overview. World J Gastrointest Oncol.

[17] Bairey O, Ruchlemer R, Shpilberg O (2006). Non-Hodgkin’s lymphomas of the colon. Isr Med Assoc J.

